# The Longitudinal Assessment of Vascular Parameters of the Retina and Their Correlations with Systemic Characteristics in Type 2 Diabetes—A Pilot Study

**DOI:** 10.3390/vision6030045

**Published:** 2022-07-20

**Authors:** Rehana Khan, Sajib K Saha, Shaun Frost, Yogesan Kanagasingam, Rajiv Raman

**Affiliations:** 1 Shri Bhagwan Mahavir Vitreoretinal Services, Sankara Nethralaya, Chennai 600006, Tamil Nadu, India; rehana.khan2293@gmail.com; 2 Australian e-Health Research Centre, The Commonwealth Scientific and Industrial Research Organisation (CSIRO), Kensington, WA 6151, Australia; sajib.saha@csiro.au (S.K.S.); shaun.frost@csiro.au (S.F.); 3 Digital Health and Telemedicine, The University of Notre Dame, Fremantle, WA 6160, Australia; yogi.kanagasingam@nd.edu.au

**Keywords:** retinal vessels, diabetes mellitus type 2, diabetic retinopathy, vascular analysis, microvascular changes

## Abstract

The aim of the study was to assess various retinal vessel parameters of diabetes mellitus (DM) patients and their correlations with systemic factors in type 2 DM. A retrospective exploratory study in which 21 pairs of baseline and follow-up images of patients affected by DM were randomly chosen from the Sankara Nethralaya–Diabetic Retinopathy Study (SN DREAMS) I and II datasets. Patients’ fundus was photographed, and the diagnosis was made based on Klein classification. Vessel thickness parameters were generated using a web-based retinal vascular analysis platform called VASP. The thickness changes between the baseline and follow-up images were computed and normalized with the actual thicknesses of baseline images. The majority of parameters showed 10~20% changes over time. Vessel width in zone C for the second vein was significantly reduced from baseline to follow-up, which showed positive correlations with systolic blood pressure and serum high-density lipoproteins. Fractal dimension for all vessels in zones B and C and fractal dimension for vein in zones A, B and C showed a minimal increase from baseline to follow-up, which had a linear relationship with diastolic pressure, mean arterial pressure, serum triglycerides (*p* < 0.05). Lacunarity for all vessels and veins in zones A, B and C showed a minimal decrease from baseline to follow-up which had a negative correlation with pulse pressure and positive correlation with serum triglycerides (*p* < 0.05). The vessel widths for the first and second arteries significantly increased from baseline to follow-up and had an association with high-density lipoproteins, glycated haemoglobin A1C, serum low-density lipoproteins and total serum cholesterol. The central reflex intensity ratio for the second artery was significantly decreased from baseline to follow-up, and positive correlations were noted with serum triglyceride, serum low-density lipoproteins and total serum cholesterol. The coefficients for branches in zones B and C artery and the junctional exponent deviation for the artery in zone A decreased from baseline to follow-up showed positive correlations with serum triglycerides, serum low-density lipoproteins and total serum cholesterol. Identifying early microvascular changes in diabetic patients will allow for earlier intervention, improve visual outcomes and prevent vision loss.

## 1. Introduction

Diabetic retinopathy (DR) is the most recognized complication of diabetes mellitus (DM). Among the working population, it is one of the most common causes of vision loss and blindness [1]. The duration of DM is the largest contributing factor to the development and severity of DR, and hence early identification of microvascular changes is essential in order to minimize visual loss and prevent the worsening of retinal damage [2,3].

In the past decade, the accurate characterization of retinal vascular changes was made possible through advances in the digital retinal photography and imaging techniques [4]. The retinal vascular geometry may represent subclinical measures of microvascular changes. These retinal vasculature changes may be a potential biomarker for future diabetes complications like DR [5,6,7,8,9]. Few epidemiologic studies extensively analysed data to find the association of retinal vessel calibre. This may provide a more global indication of the overall health of the vascular system [10]. Various population-based clinical studies have reported that retinal microvascular structural change is associated with systemic diseases [11,12]. Additionally, the changes in retinal vascular features like tortuosity and branching angle provides micro-vascular understanding of organs like kidney, brain, and heart and may be predictive of clinical vascular events [13].

There are scarce data regarding longitudinal changes in retinal vascular parameters over time and the factors influencing these changes. Among adults with type 1 and type 2 diabetes aged 40+ years from the Wisconsin Epidemiologic Study of Diabetic Retinopathy [6] mean arteriolar calibres narrowed slightly and mean venular calibres widened with increasing age. Liew et al. [14] also demonstrated that retinal vessels dilated and became more tortuous over the follow-up period of 2.6 years in type 1 diabetes. A few studies found that there is no association with the vascular measurements [15,16], while others showed that the fractal dimension of the vessels depends on the severity of the disease [17]. These inconsistent findings stem from differences in type of diabetes, study design and population ethnicity across studies. Identifying early microvascular changes in patients with diabetes will facilitate earlier intervention and treatment, improving visual outcomes and preventing further vision loss in patients with diabetic retinopathy.

This study assesses the retinal vascular parameters in patients with diabetes in order to identify which characteristics, if any, could be used as an early predictor of DR and its correlations with systemic characteristics.

## 2. Materials and Methods

### 2.1. Study Population

The 21 patients included in the study were taken from a population based four-year follow-up dataset (Sankara Nethralaya–Diabetic Retinopathy Epidemiology and Molecular Genetic Study (SN DREAMS I and II). The study design and research methodology of SN DREAMS I and SN DREAMS II have been described elsewhere [18,19]. To summarize, the Sankara Nethralaya–Diabetic Retinopathy Epidemiology and Molecular Genetic Study (SN DREAMS) was conducted between 2003 and 2005, and the cohort was followed up after 4 years (SN-DREAMS II), which was between 2007 and 2010. The study was carried out in the city of Chennai, Tamil Nadu, which is divided into 10 corporation zones of 155 divisions. This study was approved by the institutional review board (ethics committee) at Vision Research Foundation, Chennai. Informed consent was waived by the ethics committee, and the study adhered to the tenets of the Declaration of Helsinki. 

People with diabetes were identified based on the World Health Organization (WHO) criteria. A detailed history, including demographics, diabetes history and complications and significant ocular history was obtained from all patients prior to comprehensive eye screening. All the subjects underwent a detailed ophthalmic evaluation that included the assessment of visual acuity and refraction, anterior segment examination, the measurement of intraocular pressure, the grading of lens opacities and fundus examination. The details of these tests are mentioned elsewhere [18,19].

### 2.2. Photographic Methods and Retinopathy Grading

The dilated fundi of all patients were photographed following a standardized protocol using a 45° four-field stereoscopic digital fundus camera (VISUCAMlite; Carl Zeiss, Jena, Germany), and an additional 30° seven-field stereo digital pairs were taken for participants with DR. The diagnosis of DR was based on the Klein classification (modified ETDRS scale). Two independent observers graded in a masked manner; the grading agreement was high (k = 0.82) [20].

Diabetic retinopathy was graded as follows: [21]

- No diabetic retinopathy—No abnormality
- Mild Non-Proliferative diabetic retinopathy (Mild NPDR)—Only microaneurysm
- Moderate Non-Proliferative diabetic retinopathy (Moderate NPDR)—More than mild, but less than severe
- Severe Non-Proliferative diabetic retinopathy (Severe NPDR)—Any of the following: 20 or more intraretinal haemorrhages in 4 quadrants, venous beading in >2 quadrants or intraretinal neovascularization in 1 quadrant
- Proliferative diabetic retinopathy (PDR)—One or more of the following: neovascularization or preretinal or vitreous haemorrhage

**Figure 1:**
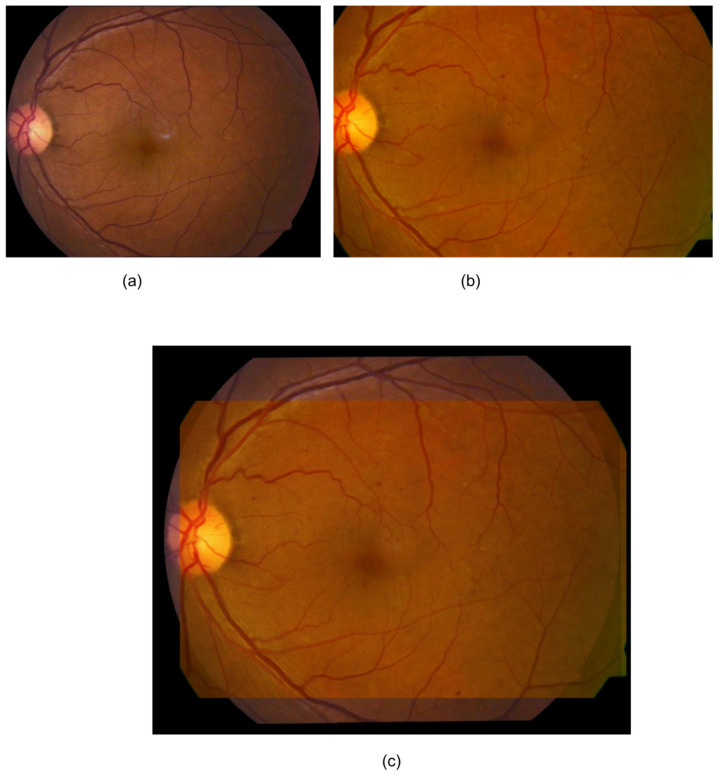
An example registration by Saha et al.’s method [23]. (**a**) Baseline image; (**b**) follow-up image; (**c**) mosaic image after registration. Only a fundus area that was common to both of the images was used for the analysis (**c**).

Changes (i.e., difference) in thickness parameters between the baseline and follow-up images were computed and finally normalized with the actual thickness of the baseline image. Prior to computing the vascular parameters, baseline and follow-up images were registered based on the vessel centreline [22], which is a process of establishing pixel-to pixel correspondence between two or more retinal images from different times, viewpoints and sources (Figure 1).

Vessel thickness parameters were generated using a web-based retinal vascular analysis platform called (VASP) [12,24]. VASP allows retinal images from various fundus cameras, pre-processes the image and performs the automated detection of the optic disc and macula. VASP also generates the vessel network from the image and identifies arteries, veins, bifurcation and cross-over points, which are dormant points through which the reference and floating images were matched distinctively. The generation of vascular parameters is a multi-stage process in VASP, and VASP allows users to interact at every single point. In this study, we have used 27 parameters for assessing retinal vascular geometry (Table 1). Statistical analyses were performed to correlate the vascular parameters with systemic parameters.

**Figure 2:**
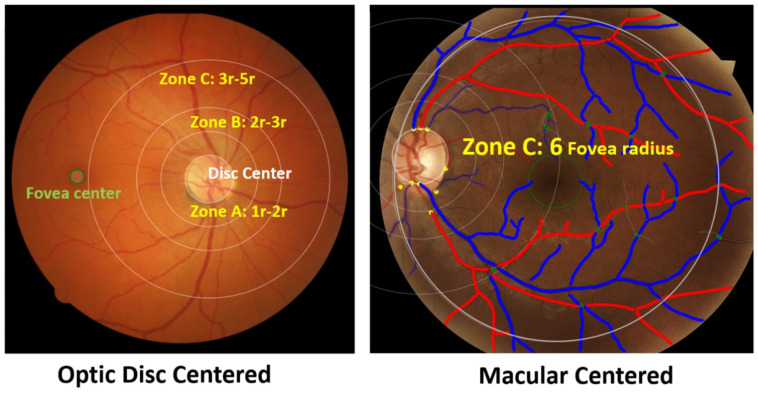
Zones in the retinal photograph. The left figure shows an example optic disc centred image, and the right figure shows an example macular centred image. The right figure also shows vessels classified into arteries and veins. Arteries are shown in red and veins are shown blue.

**Figure 3:**
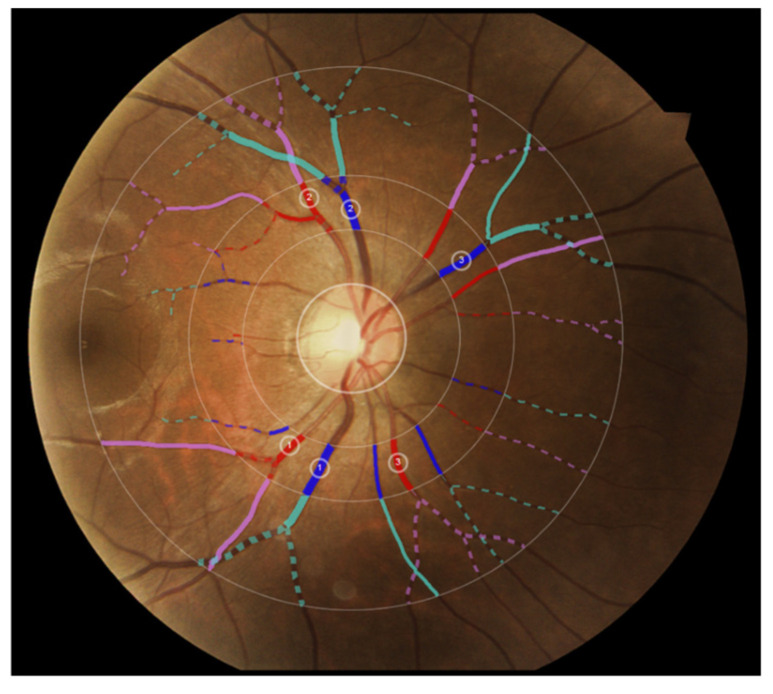
Example demonstration of the six major vessels (three arteries and three veins) used in computing VASP parameters. Vessels in different zones are represented in different colours for illustration purposes.

Figure 2 shows the three zones (i.e., zone A, zone B and zone C) that were used in computing VASP parameters. Each zone represents a circular area defined by the radius with respect to the optic disc centre. Figure 3 shows an example demonstration of the six major vessels (three arteries and three veins) that were used in computing VASP parameters.

### 2.3. Statistical Analysis

Statistical analyses were performed using SPSS (version 21.0). Mean and standard deviation were presented for continuous variables. Categorical data were represented as a number (percentage). Mean values were compared using Student t-tests, and chi-square test was performed for the categorical data to compare the distribution of the sample at baseline and follow-up and to compare the differences in vessel parameters at baseline and 4-year follow-up. For those parameters which had statistically significant difference in the two time points were further analysed for finding correlation with systemic characteristics using Pearson correlation test. Statistical significance was considered if the *p* value is less than 0.05.

## 3. Results

Table 2 shows the patient’s characteristics at baseline and at four-years follow-up. Of the various parameters, diastolic pressure shows a significant change from baseline 88.00 ± 12.65 mmHg to follow-up 77.33 ± 9.61 mmHg, (*p* value—0.004). Also on 4-year follow-up, 26.6% participants developed neuropathy (*p* = 0.012).

The majority of vascular parameters measured by VASP showed about 10~20% changes over four years’ time. However, these changes do not show any consistent pattern. Only a few parameters, namely, equiwidth3D_V_, equiwidth3D_V_2, Thicktort and Thicktort_A_ showed a reducing trend with few outliers in the four years follow-up.

Table 3 indicates the differences in parameters at baseline and 4-year follow-up. Those parameters which had statistically significant differences at the two time points were further analysed for finding correlations with the systemic characteristics.

### 3.1. Width Parameters

The equivalent vessel width in zone C for the second vein was significantly reduced from baseline (130.46 ± 31.94) to follow-up (118.16 ± 30.25) (*p* = 0.000), which showed positive correlations with systolic blood pressure (r = 0.621, *p* = 0.014) and serum high-density lipoproteins (r = 0.666, *p* = 0.007). Other width parameters did not have any correlation with systemic characteristics.

### 3.2. Fractal Dimension

The fractal dimension for all vessels in zones B and C showed a minimal increase from baseline (1.25 ± 0.07) to follow-up (1.28 ± 0.07) (*p* = 0.002. Positive correlations were noted with diastolic pressure (r = 0.621, *p* = 0.013), mean arterial pressure (r = 0.539, *p* = 0.038), serum triglycerides (r = 0.670, *p* = 0.006), serum low-density lipoproteins (r = 0.708, *p* = 0.003) and total serum cholesterol (r = 0.760, *p* = 0.001).

The fractal dimension for the vein in zones A, B and C also showed a minimal increase from baseline (1.09 ± 0.10) to follow-up (1.12 ± 0.11) (*p* = 0.014. It showed strong correlation with mean arterial pressure (r = 0.559, *p* = 0.30). However, other fractal dimension parameters did not have any correlation with the systemic characteristics.

### 3.3. Lacunarity

Lacunarity for all vessels in zones A, B and C showed a significant decrease from baseline (0.52 ± 0.14) to follow-up (0.51 ± 0.13), *p* = 0.003. Negative correlation was noted with pulse pressure (r = −0.549, *p* = 0.042). Lacunarity of the vein in zones A, B and C was slightly changed from baseline (0.58 ± 0.24) to follow-up (0.58 ± 0.15), *p* = 0.015. A positive correlation was noted with serum triglycerides (r = 0.630, *p* = 0.028).

### 3.4. Central Reflex

The vessel width for the first artery significantly increased from baseline (46.05 ± 53.19) to follow-up (75.90 ± 43.71) (*p* = 0.052). It showed positive correlations with high-density lipoproteins (r = 0.495, *p* = 0.061) and glycated haemoglobin A1C (r = 0.544, *p* = 0.036). The central reflex intensity ratio for the second artery decreased significantly from baseline (0.86 ± 0.55) to follow-up (0.19 ± 1.02) (*p* = 0.038. Positive correlations were noted with serum triglycerides (r = 0.608, *p* = 0.021), serum low-density lipoproteins (r = 0.587, *p* = 0.027) and total serum cholesterol (r = 0.639, *p* = 0.014). The vessel width for the second artery was significantly increased from baseline (9.83 ± 29.14) to follow-up (27.64 ± 36.99) (*p* = 0.057). It showed positive correlations with serum low-density lipoproteins (r = 0.486, *p* = 0.066), and total serum cholesterol (r = 0.453, *p* = 0.090).

### 3.5. Branch Parameters

The number of all trees with branches in zones B and C increased from baseline (4.60 ± 1.96) to follow-up (5.47 ± 2.75) (*p* = 0.043). This showed a positive correlation with serum low-density lipoproteins (r = 0.482, *p* = 0.069).

The branch coefficients in zones B and C slightly decreased from baseline (1.41 ± 0.57) to follow-up (1.20 ± 0.53), *p* value—0.035. It showed a positive correlations with serum triglycerides (r = 0.668, *p* = 0.025), serum low-density lipoproteins (r = 0.556, *p* = 0.076) and total serum cholesterol (r = 0.563, *p* = 0.071).

The junctional exponent deviation for artery in zone A significantly decreased from baseline (0.31 ± 0.69) to follow-up (0.0005 ± 0.52), *p* = 0.033. It showed positive correlations with serum triglycerides (r = 0.621, *p* = 0.041), serum low-density lipoproteins (r = 0.594, *p* = 0.054), and total serum cholesterol (r = 0.613, *p* = 0.045).

## 4. Discussion

This web-based retinal vascular analysis platform allows for visualizing the retinal vasculature and for the accurate assessment of vascular changes in the retina. In this article, we have discussed vascular changes from the baseline to 4-year follow-up. Our study provides information on the quantitative measurement of vascular parameters of the retina and their associations with systemic factors in a cohort of people with diabetes. Vascular parameters like width, lacunarity of the vessels, central reflex intensity ratio, branch coefficients and junctional exponent deviation for the artery decreased from baseline to follow-up, whereas fractal dimension, vessel width of the central reflex and number of branches increased from baseline follow-up. The factors independently correlating with changes in vascular parameters included diastolic and mean blood pressure for an increase in fractal dimension in the vein in zones A, B and C; diastolic blood pressure for an increase in the number of all trees with branches in zones B and C and pulse pressure for a decrease in the junctional exponent deviation for artery in zone A.

In our study, we found that the equivalent vessel width in zone C for the second vein decreased from baseline to follow-up. Similarly, Gerald Liew et al. [25] showed that both arteriolar and venular calibres narrow with age and also reported that increased vessel stiffness is due to hypertension and cardiovascular diseases. Another study [26] which included both type 1 and type 2 diabetic adults showed that the mean arteriolar calibres narrowed slightly, while the mean venular calibres widened with increasing age. Venular diameter is also reported to be narrow in people with increased blood pressure, and smoking was related with wider arteriolar calibre [25].

We found that the junctional exponent deviation for the artery in zones B and C narrowed from baseline to follow-up. Similarly, in a retrospective observational study [27], bifurcation angles narrowed with an increase in blood pressure. Roxanne Crosby-Nwaobi et al. [28] showed in PDR patients a significant change in the junctional exponential factor of the arteries (*p* = 0·010), whereas veins did not show much of deviation (*p* = 0.460). Another study by Sasongko et al. [29] found a larger arteriolar branching angle in patients with increased diabetes duration. A few studies reported that prominent blood flow with low consumption of energy will have an optimal branching angle [30,31]. Another study showed that the efficiency of branching angle depends on the size of the parent vessel [29]. Those authors found an impairment of the branching angle and junctional exponent atherosclerosis, blood flow alteration and endothelial dysfunction [32,33]. Another study showed that the oxygen saturation was reduced in case of increased branching angle [34].

We reported the central reflex intensity ratio for the second artery, which decreased from baseline to follow-up. A similar finding was noted by Brinchmann-Hansen el al [35], who noted that the intensity of light incident from the fundus photography is reflected less in the retinal arteries and veins of diabetic eyes than in the control eyes, and even the reflexes in the veins of the diabetic eyes were narrower than the control group.

Lacunarity analysis is used to measure the gaps between binary images with the same fractal dimensions, which will appear as a change in the anatomical structures [36,37,38,39]. Though it will not distinguish the vascular network geometry compared with the fractal dimension, it can be used for vascular assessment in diabetic retinopathy and macular oedema [40,41]. Differences in pixel density of varying box size and orientation of grid were determined as lacunarity (*λ*) in this study. We found that the lacunarity for all vessels in zones A, B and C decreased from baseline to follow-up and the fractal dimension for all vessels in zones B and C increased. Leontidis et al. [40] found an inverse correlation in which the lacunarity of the retinal vascular network decreased as the fractal dimension increased. Previous studies on diabetes showed a reduction in the fractal dimension [40,41,42].

We found that the fractal dimension for the vein in zones A, B and C correlated independently with changes in diastolic and mean blood pressure. Another study reported that the fractal dimension was associated with systolic, diastolic, mean arterial and pulse pressure [42,43]. Compared with the association between central retinal arteriolar equivalent (CRAE) and systolic blood pressure, the fractal dimension showed a stronger association with blood pressure [44]. For the severity of hypertension, the fractal dimension has better sensitivity than CRAE.

Previous studies with fluorescein angiograms and red-free images have reported that age is associated with decrease in junctional exponents with not much differences normotensive and hypertensive subjects [27,34]. However, in our study, we found that the pulse pressure was strongly correlated with a decrease in the junctional exponent deviation for artery in zone A. Changes in pulse pressure in diabetes reflect the stiffness of the vessels, which results in a reduction of junctional exponent deviation.

The strengths of the study were the prospective design, that a standard protocol was used to evaluate the retinal images and that a web-based program was used to quantitatively examine the retinal vasculature. Our study has a few limitations. Firstly, it uses a limited sample size. Second, the smaller retinal vessels might have been missed due to poor resolution of the fundus photographs. Thirdly, there was less precision in the measurement of a few parameters like the contrast and brightness of images in spite of following the standard protocols. Lastly, refractive errors like high myopia may have some correlation to retinal vessels, and hence, future studies should have control groups without diabetes to see if the 4-year changes were due to diabetes or other effects.

Information processing and contact with the environment, as well as the performance of daily activities, are all aided by vision. Visual impairment has been linked to a reliance on assistance with daily tasks, social isolation and decreased physical activity. In this regard, people with DR’s visual impairment and its major impacts on several aspects of their lives can result in a significant decline in their quality of life [45].

## 5. Conclusions

In conclusion, there were parameters which changed over time in diabetes. There is clearly a change in the retinal vascular features and pattern which was caused by the effects of diabetes. Changes in retinal vascular calibre reflect a variety of subclinical pathophysiologic responses to hyperglycaemia, hypertension and dyslipidaemia and can predict not just various diabetic microvascular issues but also stroke and coronary heart disease. Longitudinal variations in retinal vascular parameters can be utilised for the early detection of diabetic complications. Positive outcomes will provide the much-desired opportunity to apply cost-effective prevention and intervention techniques. It is important to include people from similar cultural, ethnic, and socioeconomic backgrounds in order to notice any differences and further evaluate the model’s performance. Similar tests on a larger dataset and with other demographics are also required.

## Figures and Tables

**Table 1:** Vessel parameters generated by VASP that are used in this study.

Vessel Thickness Parameters	Description
equiAVR_B_	Vessel width ratio for zone B
equiWidth3B_A_	Width ratio of artery for zone B
equiWidth3B_V_	Width ratio of vein for zone B
equiAVR_B_2	Width ratio for zone B (computed using a different algorithmic approach than ‘equiAVR_B_’)
equiWidth3B_A_2	Width ratio of artery for zone B (computed using a different algorithmic approach than ‘equiWidth3B_A_’)
equiWidth3B_V_2	Width ratio of vein for zone B (computed using a different algorithmic approach than ‘equiWidth3B_V_’)
equiAVR_C_	Width ratio for zone C
equiWidth3C_A_	Width ratio of artery for zone C
equiWidth3C_V_	Width ratio of vein for zone C
equiAVR_C_2	Width ratio for zone C (computed using a different algorithmic approach than ‘equiAVR_C_’)
equiWidth3C_A_2	Width ratio of artery for zone C (computed using a different algorithmic approach than ‘equiAVR_C_’)
equiWidth3C_V_2	Width ratio of vein for zone C (computed using a different algorithmic approach than ‘equiWidth3C_V_’)
equiAVR_D_	Width ratio for zone D
equiWidth3D_A_	Width ratio of artery for zone D
equiWidth3D_V_	Width ratio of vein for zone D
equiAVR_D_2	Width ratio for zone D (computed using a different algorithmic approach than ‘equiAVR_D_’)
equiWidth3D_A_2	Width ratio of artery for zone D (computed using a different algorithmic approach than ‘equiWidth3D_A_’)
equiWidth3D_V_2	Width ratio of vein for zone D (computed using a different algorithmic approach than ‘equiWidth3D_V_’)
SimTort	Simple tortuosity for all vessels in zones B and C
CurvTort	Curvature tortuosity for all vessels in zones B and C
ThickTort	Thick tortuosity for all vessels in zones B and C
SimTort_A_	Simple tortuosity for artery in zones B and C
CurvTort_A_	Curvature tortuosity for artery in zones B and C
ThickTort_A_	Thick tortuosity for artery in zones B and C
SimTort_V_	Simple tortuosity for vein in zones B and C
CurvTort_V_	Curvature tortuosity for vein in zones B and C
ThickTort_V_	Thick tortuosity for vein in zones B and C

**Table 2:** The basic characteristics of the subjects at baseline and at follow-up.

Basic Characteristics	Base Line	Follow-Up	*p* Value
Age in years (Mean ± SD)	49.73 ± 4.76	53.73 ± 4.76	0.010
Gender			
Male, N (%)	9 (60)	9 (60)	-
Female, N (%)	6 (40)	6 (40)	
BCVA (Log MAR), (Mean ± SD)	0.04 ± 0.21	0·04 ± 0.21	-
Cataract			
Present, N (%)	0 (0)	1 (6.66)	0.235
Absent, N (%)	15 (100)	14 (93.33)	
BMI (kg/m^2^), (Mean ± SD)	27.37 ± 5.59	26.52 ± 5.29	0.616
FBS (mg/dL), (Mean ± SD)	140.07 ± 43.54	161.87 ± 57.33	0.173
HbA1c (%), (Mean ± SD)	6.96 ± 0.98	7.58 ± 0.99	0.052
Systolic Blood Pressure (mmHg), (Mean ± SD)	135.73 ± 14.20	126.00 ± 17.65	0.056
Diastolic Blood Pressure (mmHg), (Mean ± SD)	88.00 ± 12.65	77.33 ± 9.61	0.004
Serum Triglycerides (mg/dL), (Mean ± SD)	120.13 ± 33.96	134.67 ± 43.88	0.237
HDL (mg/dL), (Mean ± SD)	34.00 ± 5.33	32.67 ± 8.40	0.544
LDL (mg/dL), (Mean ± SD)	122.71 ± 25.60	122.07 ± 27.95	0.939
Serum total Cholesterol (mg/dL), (Mean ± SD)	180.73 ± 27.30	193.80 ± 31.30	0.157
Nephropathy			
Present, N (%)	2 (13.33)	4 (26.66)	0.286
Absent, N (%)	13 (86.66)	11(73.33)	
Neuropathy			
Present, N (%)	0(0)	4(26·66)	0.012
Absent, N (%)	15(100)	11(73.33)	
Smoking Status			
Yes (%)	1 (6.66)	1 (6.66)	-
No (%)	14 (93.33)	14 (93.33)	
Alcohol Status			
Yes (%)	1 (6.66)	1 (6.66)	-
No (%)	14 (93.33)	14 (93.33)	

BCVA: Best Corrected Visual Acuity; BMI: Body Mass Index; FBS: Fasting Blood sugar; HDL: High-density Lipoprotein; LDL: Low-density Lipoprotein; SD: Standard Deviation.

**Table 3:** The differences in parameters at baseline and 4-year follow-up.

Parameters		Base Line	Follow-Up	*p*
		(Mean ± SD)	(Mean ± SD)	
	Disc Radius	78.00 ± 8.42	77.87 ± 7.96	0.884
	Disc Centre_row	305.86 ± 50.47	305.00 ± 49.46	0.421
	Disc Centre_col	500.93 ± 430.76	500.20 ± 430.64	0.587
	Fovea Centre_row	365.20 ± 61.55	367.07 ± 55.29	0.707
	Fovea Centre_col	466.27 ± 73.84	481.47 ± 62.15	0.309
	Disc Zone C_IOU	0.46 ± 0.03	0.46 ± 0.03	0.982
	MCZone3_IOU	0.81 ± 0.12	0.82 ± 0.05	0.699
	Equi AVR_C	0.85 ± 0.17	0.92 ± 0.21	0.05
	equiWidth_3C_V	146.81 ± 31.32	140.52 ± 25.65	0.037
	equiAVR_C2	0.84 ± 0.16	0.92 ± 0.22	0.054
	equiWidth_3C_V2	148.61 ± 32.48	142.04 ± 26.69	0.030
	equiWidth_3D_V	130.24 ± 31.74	117.63 ± 29.70	0.000
	equiWidth_3D_V2	130.46 ± 31.94	118.16 ± 30.25	0.000
	Width Gradient_Inf_A	0.001 ± 0.01	0.003 ± 0.01	0.057
	Width Gradient_A	0.001 ± 0.01	0.005 ± 0.004	0.046
Fractal Dimension	Fractal Dim	1.25 ± 0.07	1.28 ± 0.07	0.002
	Fractal Dim_V	1.09 ± 0.10	1.12 ± 0.11	0.014
	Curv Tort_A	0.0003 ± 0.0001	0.0002 ± 0.0001	0.032
	Thick Tort_V	4.69 ± 17.24	0.01 ± 0.04	0.31
Lacunarity	Lac	0.52 ± 0.14	0.51 ± 0.13	0.003
	Lac_ A	0.61 ± 0.22	0.60 ± 0.25	0.028
	Lac_ V	0.58 ± 0.24	0.58 ± 0.15	0.015
Central Reflex	CR_Ratio_A1	0.36 ± 0.72	0.08 ± 0.57	0.033
	CR_InRatio_A1	0.08 ± 1.02	0.60 ± 0.83	0.014
	CR_VesWidth_A1	46.05 ± 53.19	75.90 ± 43.71	0.052
	CR_InRatio_A2	0.86 ± 0.55	0.19 ± 1.02	0.038
	CR_VesWidth_A2	9.83 ± 29.14	27.64 ± 36.99	0.057
Branch Parameters	Num Br	4.60 ± 1.96	5.47 ± 2.75	0.043
	Branch Coef_A	1.41 ± 0.57	1.20 ± 0.53	0.035
	JED_A	0.31 ± 0.69	0.0005 ± 0.52	0.033

## Data Availability

The datasets generated during and/or analysed during the current study are not publicly available, as it is against the organization/hospital (Vision Research Foundation, Chennai) policy. All anonymized data available upon request and are being stored in Vision Research Foundation office.
